# Rabbit haemorrhagic disease (RHD) and rabbit haemorrhagic disease virus (RHDV): a review

**DOI:** 10.1186/1297-9716-43-12

**Published:** 2012-02-10

**Authors:** Joana Abrantes, Wessel van der Loo, Jacques Le Pendu, Pedro J Esteves

**Affiliations:** 1 CIBIO/UP, Centro de Investigacao em Biodiversidade e Recursos Geneticos/Universidade do Porto, Campus Agrario de Vairao, 4485-661 Vairao, Portugal; 2 INSERM, U892, Université de Nantes, 44007 Nantes, France; 3 CITS, Centro de Investigacao em Tecnologias de Saude, CESPU, Gandra, Portugal

## Abstract

Rabbit haemorrhagic disease virus (RHDV) is a calicivirus of the genus *Lagovirus *that causes rabbit haemorrhagic disease (RHD) in adult European rabbits (*Oryctolagus cuniculus*). First described in China in 1984, the virus rapidly spread worldwide and is nowadays considered as endemic in several countries. In Australia and New Zealand where rabbits are pests, RHDV was purposely introduced for rabbit biocontrol. Factors that may have precipitated RHD emergence remain unclear, but non-pathogenic strains seem to pre-date the appearance of the pathogenic strains suggesting a key role for the comprehension of the virus origins. All pathogenic strains are classified within one single serotype, but two subtypes are recognised, RHDV and RHDVa. RHD causes high mortality in both domestic and wild adult animals, with individuals succumbing between 48-72 h post-infection. No other species has been reported to be fatally susceptible to RHD. The disease is characterised by acute necrotising hepatitis, but haemorrhages may also be found in other organs, in particular the lungs, heart, and kidneys due to disseminated intravascular coagulation. Resistance to the disease might be explained in part by genetically determined absence or weak expression of attachment factors, but humoral immunity is also important. Disease control in rabbitries relies mainly on vaccination and biosecurity measures. Such measures are difficult to be implemented in wild populations. More recent research has indicated that RHDV might be used as a molecular tool for therapeutic applications. Although the study of RHDV and RHD has been hampered by the lack of an appropriate cell culture system for the virus, several aspects of the replication, epizootology, epidemiology and evolution have been disclosed. This review provides a broad coverage and description of the current knowledge on the disease and the virus.

## Table of contents

1. Natural history

2. Aetiological agent

3. Clinical signs and lesions

4. Epidemiology

5. Virus life cycle

6. Mechanisms of resistance to RHD

7. Genetic diversity/RHDV evolution

7.1. Pathogenic RHDV

7.2. Non-pathogenic rabbit calicivirus

8. Host-virus co-evolution

9. Prevention, control and vaccination

10. Therapeutic applications of RHDV

11. Conclusions

12. List of abbreviations

13. Competing interests

14. Authors' contributions

15. Acknowledgements

16. References

## 1. Natural history

In the 1980s, the European rabbit populations were devastated by a new viral disease characterised by being extremely lethal and highly contagious in both domestic and wild rabbits (*Oryctolagus cuniculus*). The first outbreak of this new disease, designated as rabbit haemorrhagic disease (RHD), was noticed in 1984 in the Jiangsu Province of the People's Republic of China within a group of commercially-bred Angora rabbits imported from Germany [1]. In less than a year, RHD killed 140 million domestic rabbits in China and spread over an area of 50 000 km^2 ^[1,2]. Korea was the next country to report RHD outbreaks which were associated with rabbit fur importation from China [3]. The disease then appeared in Europe and was first reported in Italy in 1986 [4] from where it spread to the rest of Europe, becoming endemic in several countries. In the Iberian Peninsula, where European rabbits originated and where they constitute a key species of the ecosystem [5], the first outbreaks date back to 1988 for Spain [6] and to 1989 for Portugal [7] and caused severe reduction of wild populations [8,9]. At the same time, domestic populations from several countries in North Africa experienced RHD outbreaks [10]. In the Americas, the first outbreaks were recorded in 1988 in Mexico following the importation of rabbit products from China [11]. Nevertheless, Mexico is currently the only country that has managed to successfully eradicate RHD with the last outbreak having occurred in 1992 [11]. This successful eradication of the disease might correlate with the absence of natural populations of wild European rabbits. North America recorded the first outbreak only in 2000 and experienced a few additional outbreaks since then [12]. As the virus spread worldwide, naturally occurring RHD outbreaks were reported in geographically distant regions, such as Cuba, Uruguay and Reunion Island [13,14].

RHD causes important economic losses in the rabbit meat and fur industry and has a significant negative ecological impact among wild rabbit populations and indirectly on its dependant predators [2,11,15,16]. In Australia and New Zealand, where the rabbit is considered an important agricultural pest, as well as a major threat to the endemic wildlife flora and fauna [17,18], rabbit haemorrhagic disease virus (RHDV) was soon considered as an agent for rabbit control [19]. In 1991, a scientific research program was initiated in laboratory under quarantine measures to assess the host specificity and efficacy of the RHDV Czech reference strain (Czech V351) as a biocontrol agent. After approval of the Australian authorities, RHDV was released in the Wardang Island in Spencer Gulf, South Australia. Despite the rigorous quarantine measures, in 1995 RHDV escaped from the island, possibly transported by insects or air currents and reached the mainland [20]. In less than two years, it became established across southern Australia. The initial spread was estimated to be 50 km per week. In some areas a reduction of more than 95% of the wild rabbit populations was observed, particularly in the more arid regions [21]. In New Zealand, after a careful investigation on the benefits and risks of introducing RHDV, the government decided not to introduce the virus [19]. The virus was later illegally introduced by landholders [22]. Posterior characterisation of the New Zealand virus showed it to be similar to the Czech V351 strain introduced in Australia suggesting that it was imported from there [23].

Nowadays, RHDV outbreaks still occur on almost all continents and cause significant mortality rates, being endemic in most parts of Europe, Asia, and parts of Africa, Australia and New Zealand. As a general trend, it seems that in areas where the European rabbit is historically present as wild populations, RHDV is also present and endemic. In contrast, in regions where the European rabbit is mainly present as a domestic or industrial animal, the occurrence of RHDV (epidemics or rare outbreaks) seems to be correlated with rabbit colony number and density.

## 2. Aetiological agent

Early efforts to classify RHDV were erratic, mostly due to its non-cultivable nature. Initially suspected to be a picornavirus [24], a parvovirus [25] and a parvo-like virus [2], it was finally assessed in the early 1990s as a member of the *Caliciviridae *family [26-30].

The International Committee on Taxonomy of Viruses (ICTV) recognises four genera in the *Caliciviridae *family: *Lagovirus, Vesivirus, Norovirus *and *Sapovirus*. Three more genera were recently proposed as part of this family: *Nabovirus *or *Becovirus *[31], *Recovirus *[32] and *Valovirus *[33], but are not yet recognised by the ICTV. Caliciviruses infect a broad range of animals, including humans, and cause a variety of diseases, such as gastroenteritis by *Norovirus *and *Sapovirus*, haemorrhagic disease by *Lagovirus*, and vesicular lesions, respiratory infections and reproductive failure by *Vesivirus*. The *Lagovirus *genus comprises both RHDV and European brown hare syndrome virus (EBHSV), a virus first detected in Sweden in the early 1980s prior to the first RHDV outbreak [34] which affects hare species (*Lepus europaeus *and *Lepus timidus*). European brown hare syndrome (EBHS) is closely related to RHD with regards to clinical signs, pathological and histopathological alterations, mortality rates, virion morphology and antigenicity, but cross-species infection and cross-species protection could not be obtained in a reproducible way. Despite the similarities, RHDV and EBHSV represent distinct agents, infecting different species although causing similar diseases [35-40].

As in other caliciviruses, RHDV virions are small sized (between 35-40 nm of diameter) and non-enveloped. The capsid, which forms the protein layer that protects the RNA molecule, is composed of 90 arch-like dimers of the capsid protein which form 32 cup-shaped depressions (*calix *in Latin for cup or chalice as the root for the family name *Caliciviridae*) arranged in a T = 3 icosahedral symmetry [41,42]. Each capsid monomer consists of a shell (S) domain which is buried and comprises the N-terminal connected by a hinge to the protruding (P) domain that encompasses the C-terminal region and is exposed on the surface [39-41,43-46]. The P domain can be further subdivided into the subdomains P1 (stem of arch) and P2 (top of arch) [47]. The subdomain P2, located at the most exposed region of the capsid, displays the greatest genetic variation. This variation is probably, at least in part, due to selection pressure because host antibodies recognise and target regions located in this subdomain [43,44,48]. In order to avoid this recognition and the inherent selective pressure, these regions tend to evolve faster [49,50] which results in an increase of the genetic variability and hence, of the antigenic variation. In addition, in noroviruses, the P2 domain has been shown to contain the carbohydrate-binding domain, contributing to several more conserved amino acids [51-53]. By analogy, this should also apply to RHDV.

Sporadically, in rabbits affected by subacute or chronic forms of RHD with long clinical courses, it is possible to detect a second type of virus particle, the RHDV core-like particles (CLP), also referred to as smooth particles or s-RHDV [35]. These particles are found in large amounts in the liver and spleen [54] and present unique characteristics when compared to RHDV particles: a smooth surface due to the lack of the cup-shaped depressions; a smaller diameter of 25-29 nm; a molecular weight of 28-30 kDa indicating that CLP correspond to the N-terminus (the buried shell domain) of the capsid; no haemagglutinating activity, most likely as the result of the absence of the C-terminus, but presenting reactivity with sera from RHDV convalescent rabbits and monoclonal antibodies directed towards the N-terminal part of the RHDV capsid [35,54-56]. CLP seem to be associated with the appearance of specific anti-RHDV IgM [54]. Indeed, these particles have been suggested to result from the degradation of the RHDV-IgM immune-complexes formed during the humoral response [54]. Although defective gene expression has been suggested to be at the genesis of CLP [55], recent data indicate that CLP directly derive from intact virions with dissociated protrusion [45].

**Figure 1:**
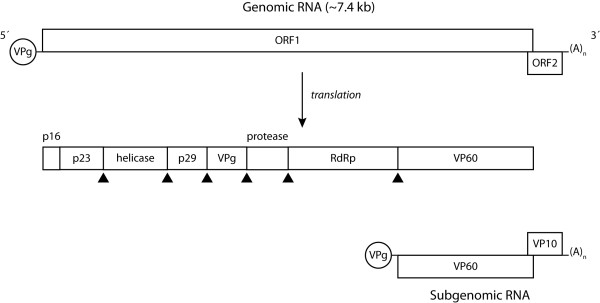
**Genomic organization of RHDV**. The genome of RHDV is composed of two narrowly overlapping ORFs, ORF1 and ORF2. ORF1 codes for a polyprotein that is cleaved by the virus-encoded trypsin-like cysteine protease (arrowheads) and originates the major structural protein for the capsid (VP60) and the non-structural proteins p16, p23, helicase, p29, VPg, protease and RdRp. ORF2 codes for a minor structural protein, VP10. A subgenomic mRNA encoding both the structural proteins VP60 and VP10 can also be found in viral particles. Both the genomic and subgenomic RNA are polyadenylated at their 3'end and have the virus-encoded protein, VPg, covalently attached to their 5' end.

RHDV virions contain the genomic RNA (gRNA) and an additional RNA species with 2.2 kb designated subgenomic RNA (sgRNA), which is collinear with the 3' end of the genomic RNA [26,57]. Subgenomic RNA usually contributes to the production of high levels of products required during the intermediate and late stages of infection (e.g. structural proteins) [58]. For RHDV, these comprise the capsid protein and VP10 [59-61]. Both the genomic and subgenomic RNA are polyadenylated at the 3' end and at their 5' region they are covalently linked through a Tyr-21 residue to the VPg (virus genome-linked) protein [62]. The genomic RNA consists of a positive-sense single-stranded molecule of 7437 nucleotides consisting of two slightly overlapping open reading frames (ORF): ORF1, comprising nucleotides 10 to 7044 and ORF2, comprising nucleotides 7025 to 7378 [26]. ORF1 encodes a large polyprotein of ca. 257 kDa [26] which is cleaved into the mature non-structural proteins and a major structural protein, the capsid protein, by post-translational proteolytic processing by a virus-encoded trypsin-like cysteine protease (Figure 1) [57,63-65]. Some of these proteins derive from larger precursors that result from further post-translational modifications of the precursor proteins [57,64]. The biological role of some of the non-structural proteins encoded by the genome of caliciviruses has been elucidated by relying on previous knowledge gathered from members of the closely related *Picornaviridae *family [26,64,65]. For RHDV, two proteins involved in the replication of the viral RNA, a helicase and an RNA-dependent RNA polymerase (RdRp), and a protease responsible for the proteolytic processing of the large polyprotein, have been characterised [63,66,67]. RdRp has been shown to also catalyse VPg uridylation [62,68] while a role in translation has been suggested for VPg [e.g. [69]]. The function of the RHDV non-structural proteins p16, p23 and p29 remains to be assessed. VP10, a minor structural protein encoded by the 3'end of gRNA and sgRNA in a different reading frame (ORF2), was recently shown to increase the levels of virus replication and to promote apoptosis [70]. In addition, its ability to downregulate the expression of VP60 was demonstrated [71]. Together, this suggests that VP10 might regulate virus replication and virion release from infected host cells [71].

## 3. Clinical signs and histopathological lesions

The incubation period of the disease ranges between 1 to 3 days and rabbits usually succumb within 12 h to 36 h after the onset of fever (> 40°C). Depending on the clinical evolution of the disease, three different clinical courses can occur [38,72]. In the peracute form, infected animals show no clinical signs and die suddenly. Acute infections are accompanied by anorexia, apathy and congestion of the palpebral conjunctiva and neurologic symptoms such as opisthotonos, excitement, paralysis and ataxia may also be observed. There are occasionally some respiratory signs (tracheitis, dyspnea and cyanosis) and a foamy and bloody nasal discharge; lacrimation, ocular haemorrhages and epistaxis can also occur. Subacute forms of the disease present similar, but milder clinical symptoms and most rabbits survive. Rabbits experiencing subacute infections develop antibodies against RHDV which confer protection upon re-infection [73]. In addition, it has been reported that during an outbreak of RHD, a low percentage of rabbits may experience a chronic form of the disease with symptoms including a severe and generalised jaundice, anorexia and lethargy [35]. These animals tend to die 1-2 weeks later [54], but animals that overcome the disease present a potent seroconversion [35]. Interestingly, this form of the disease has been shown to be associated with the presence of RHDV core-like particles [35,55].

The liver, lung and spleen are the primary target tissues of RHDV. The major histopathological lesions found at necropsy are acute hepatitis due to liver cell loss as the result of RHDV-induced apoptosis, and splenomegaly [74,75]. Haemorrhages and congestions can be seen in several organs, particularly in the lungs, heart and kidneys, as a result of a massive disseminated intravascular coagulation (DIC) which is usually the cause of death [76]. Depletion of both B and T lymphocytes in the liver and the spleen accompanies the disease and accounts for an impairment of the immune response [72,77] and a fatal progression of the disease within 2-3 days. In contrast, resistant rabbits develop high titres of IgM (and then of IgA and of IgG) already at day 3 pi, thus presenting an effective humoral immune response [54]. Table 1 presents a summary of the histopathological alterations that can be observed upon RHDV infection.

**Table 1:** Pathological and histopathological lesions [16,77,222,241,243]

Organ	Lesions
Liver	Enlarged with marked lobular pattern, yellow-grey colour, brittle, circumscribed infiltration with granulocytes, degenerative alterations of hepatocytes compatible with apoptosis (extensive vacuolization, severe alterations in the mitochondrial structure, karyopyknosis and karyolysis) activation of Kupffer cells, leukopenia

Trachea	Hyperaemia of mucous membrane, petechial or diffuse haemorrhages, may be filled with bloody foam

Lung	Hyperaemia, pulmonary oedema, intra-alveolar and perivascular haemorrhages, sometimes slight catarrhal bronchiolitis, proliferation of lymphocytes

Kidneys	Enlargement with spotted dark red coloration, hyperaemia, haemorrhages within glomerular loops and renal medulla, hyaline thrombi, dilated tubuli, lymphocytic infiltration, degeneration of tubular epithelium

Spleen	Enlargement (splenomegaly), spotted dark red colorations, hyperaemia, occasionally karyorrhexis within follicles, hemosiderosis, leukopenia

Digestive tract	Contents usually normal, occasional enteritis, subserous haemorrhages

Chest and abdominal cavity	Small amounts of serous, occasionally bloody exudate, sometimes subserous haemorrhages

Muscles	Anaemia in the area of the thighs, petechiae in the heart muscle, focal necrosis in myocardium, degenerative alterations, hemosiderosis

Central Nervous System	Congestion of cortical vessels, dilated vessels in the area of the pia mater of the cortex and cerebellum, hyperaemia, small haemorrhages in the cortex, occasionally non-purulent encephalomyelitis with lymphocytic infiltration

## 4. Epidemiology

The possible routes for transmission of the disease are the oral, nasal, conjunctival and parenteral, as blood-feeding insects have also been shown to be efficient mechanical vectors [72,78]. Transmission of RHDV may occur through direct contact with an infected animal, since infected rabbits may shed viral particles in their secretions and excretions [79], or indirectly by means of fomites-contaminated food, bedding, water, clothing, cages and equipment [19]-or vector-borne transmission by scavenging mammals, birds and insects [e.g. [78,80,81]]. The natural doors for viral entry have been suggested to be located in the upper respiratory and digestive tract [16,82]. In natural infections, the faecal-oral route is considered the preferential mode of transmission [10,11].

In the field, carcasses of RHDV-infected rabbits may be a major source for viral spreading since the virus seems to be highly resistant and stable when exposed to harsh environmental conditions. Indeed, carcasses of RHDV-infected rabbits exposed to environmental conditions have been found to contain viable viral particles for up to three months [83,84]. This ability is paramount for the epidemiology of RHD and supports the importance of indirect routes in transmission. Environmental factors have also been suggested to impact on the effectiveness of RHD in rabbit populations [reviewed in [20]]. Temperature and humidity seem to be the most important climate variables. Indeed, in Australia, mortality rates due to RHD are higher in arid and semi-arid inland areas than in moist coastal regions experiencing milder temperatures and the disease becomes active during the breeding season, peaks in early spring and is absent in the summer [85]. Climate variables might contribute to the geographic and seasonality observed for the RHDV outbreaks by affecting the abundance and activity of the vectors involved in RHDV transmission [reviewed in [20]]. Other non-climatic factors have also been suggested to contribute to the variable pattern of the impact of RHD in rabbit populations such as the timing of the breeding season, the presence of a related and protective RHDV-like calicivirus in rabbit populations or the negative interaction of the myxomatosis outbreaks in the populations [reviewed in [86]]. In addition, modelling studies indicated that population dynamics and spatial structure may greatly influence disease impact and host-virus co-evolution [87,88].

Caliciviruses occur in a wide range of animals apart from rabbits, which include mustelids (minks and skunks), reptiles, cattle, felids (cats and cheetahs), dogs, humans, chimpanzees, pigs and sea mammals (sea lions, seals, walrus, whales, and dolphins), but they are usually restricted to their primary host and closely related species [89]. Indeed, rabbits and hares are the only hosts for the RHDV and EBHSV lagoviruses, respectively. Other leporid species have been shown not to be susceptible to RHDV [11]. Additionally, several non-host species from the Australian fauna, including domestic, feral animals and wildlife, were assessed for susceptibility to RHDV. No viral replication could be detected with an extensive panel of tests which included clinical observations, pathology, electron microscopy, virology and serology, reinforcing the idea that susceptibility to RHDV is restricted to the European rabbit (*Oryctolagus cuniculus*) [90]. Both subspecies of the European rabbit, *O. c. cuniculus *and *O. c. algirus*, seem equally susceptible to RHDV [91]. Interestingly, antibodies against RHDV had been found in animals that live in sympatry with rabbit populations infected with RHDV [81,92,93] and, more recently, RHDV RNA was isolated from sympatric wild micromammals opening the possibility of other species being involved in the epidemiology of the disease [94].

## 5. Virus life cycle

In adult rabbits, the targets of the initial stages of the virus life cycle have been determined. Indeed, viral antigens are detected in the liver within the first hours following infection with RHDV with viral replication occurring in the cytoplasm of hepatocytes located mostly in centriacinar areas [30,74,95-98]. The number of infected hepatocytes clearly increases in the course of the disease, reaching a maximum between 36-48 h [95-97]. Detection of viral antigens in Kupfer cells has also been reported [74,98] associated with viral replication [98]. Extrahepatic presence of the virus has also been observed, but some discrepancies exist between the different studies since different techniques had been employed. Nevertheless, viral antigens have been detected in the spleen, in particular in the macrophages located in the red pulp [96-98], kidney [96], and alveolar macrophages in the lungs. It has been suggested that the presence of replicating virus in alveolar macrophages, which are in contact with the bloodstream, might be important for initial virus dissemination, and later when the virus reaches the liver, Kupfer cells may be important for spreading the infection into other organs [98].

In contrast, viral dissemination in young resistant rabbits is far unclear. Viral antigens have been detected in hepatocytes from experimentally-infected 2-week old rabbits [99], but most studies were only able to detect them in rabbits older than 4-weeks [96,100]. Viral antigens were found to be scattered and present in only a small percentage of cells. Nevertheless, this suggests that some hepatocytes in young (resistant) rabbits are able to support viral replication, but that major changes must occur in the liver to support a full infectious process. In addition, clearance of the virus seems to be extremely rapid as no viral antigens were detected after day 4 pi [100]. The presence of the virus in other organs has not been fully assessed.

**Figure 2:**
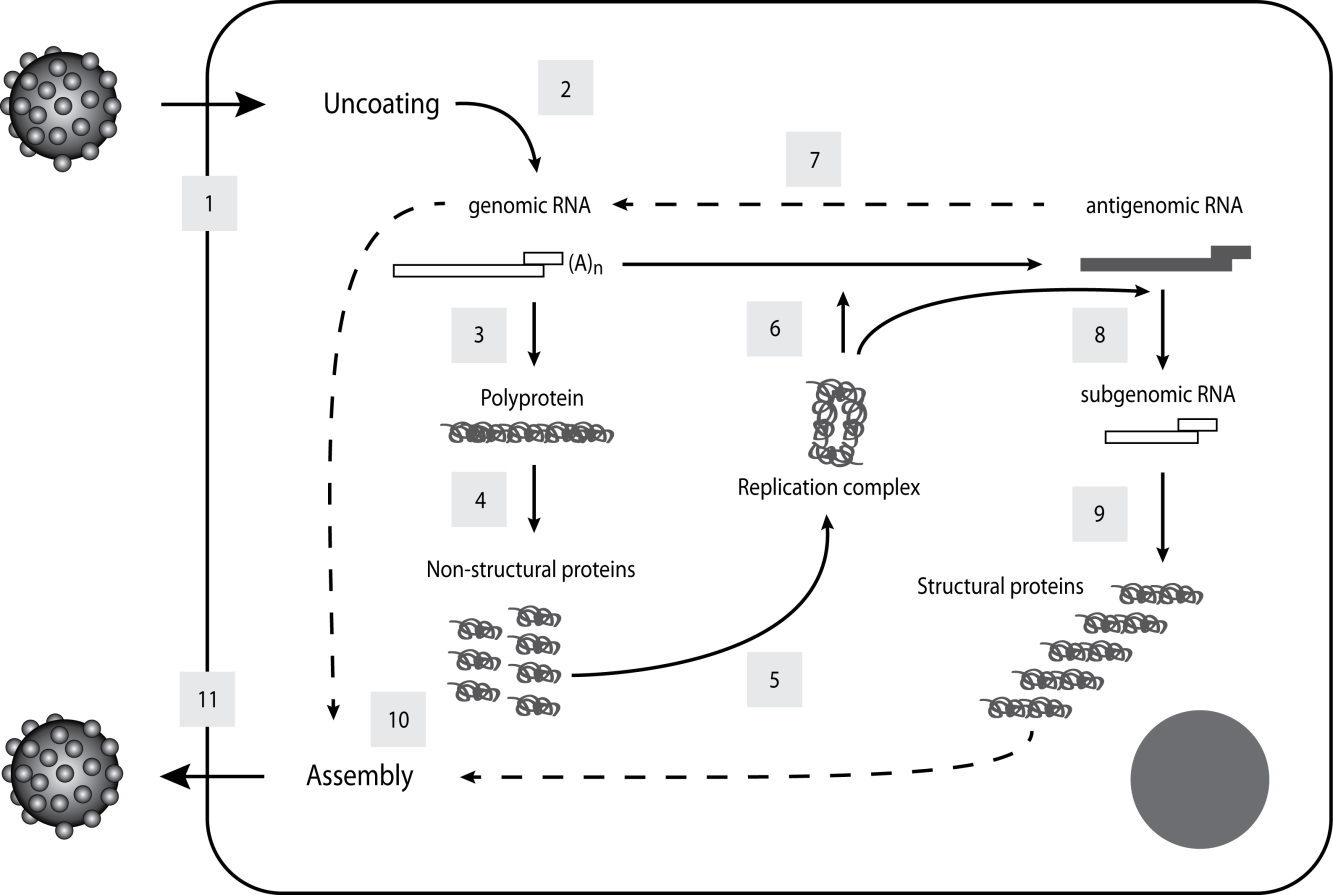
**The replication cycle of caliciviruses**. After attachment to the cellular receptor, the virion is internalised into the cell (step 1). Uncoating of the viral genome (step 2) is followed by translation of the polyprotein precursor (step 3) and co-translational processing releasing the non-structural proteins (step 4). These proteins assemble in a replication complex (step 5) that synthesises the antigenomic RNA (step 6), being itself used as a template for synthesis of the genomic RNA (step 7). The newly synthesized genomic RNA is translated as a polyprotein precursor (step 3) or is used for packaging in the assembled viral protein core (step 10). The antigenomic RNA is also the template for synthesis of subgenomic RNA (step 8). The subgenomic RNA is translated as structural proteins, VP60 and VP10 (step 9) and in lagoviruses, VP60 is also released from the polyprotein precursor after processing by the viral protease. At a still not defined time in the virus life cycle, assembly of the structural proteins as well as packaging of the genomic RNA occurs (step 10), followed by release of the mature virion from the cell (step 11). Reprinted from Antiviral Research, 87, Rohayem J, Bergmann M, Gebhardt J, Gould E, Tucker P, Mattevi A, Unge T, Hilgenfeld R, Neyts J, Antiviral strategies to control calicivirus infections, 167, 2010, with permission from Elsevier.

**Figure 3:**
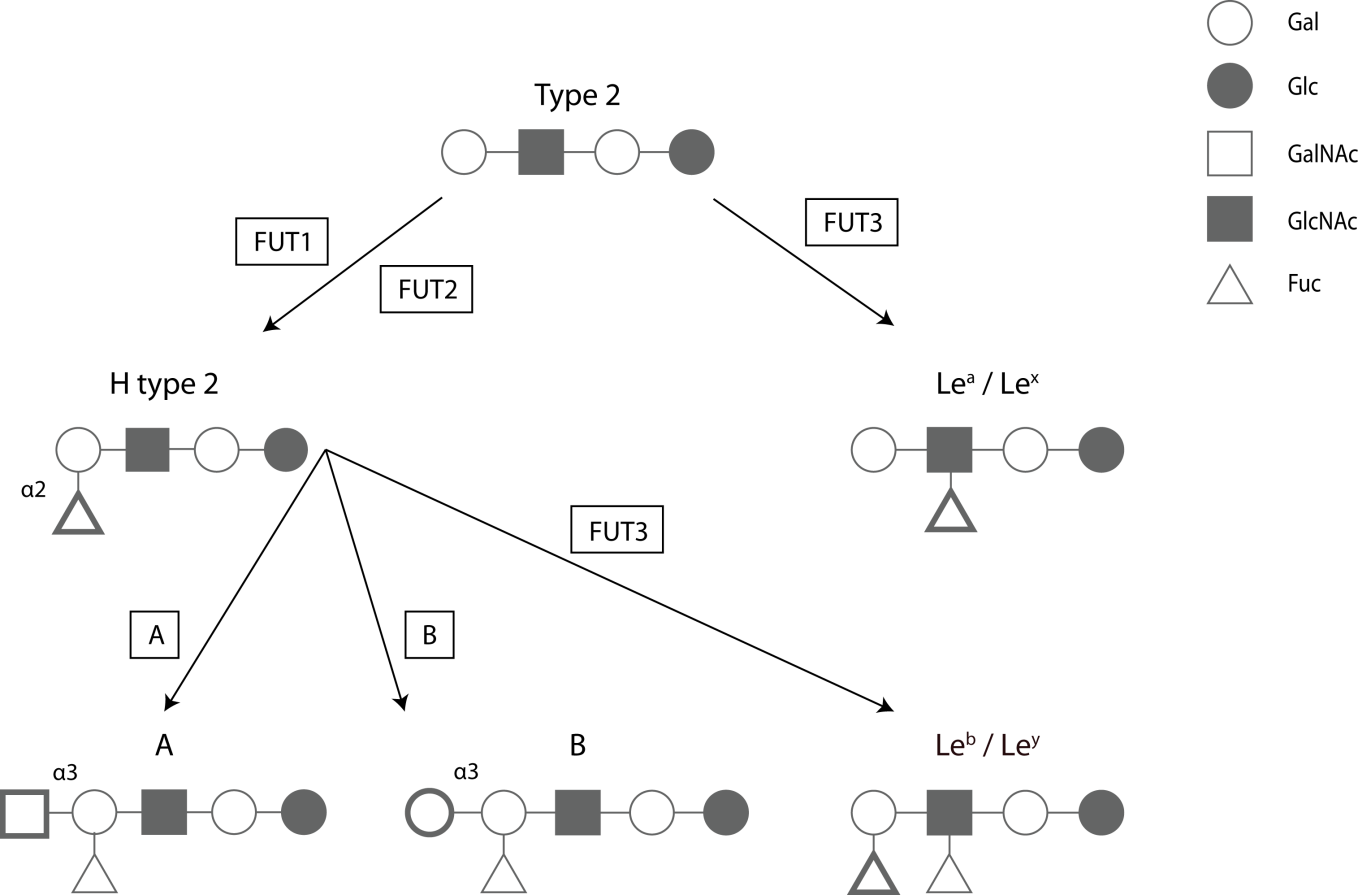
**Schematic biosynthesis of the HBGA ABH and Lewis**. Several transferase enzymes (boxed) are involved in the addition of relevant monosaccharides (in bold) to synthesise the ABH and Lewis ligands in a variety of tissues. Gal, Galactose; Glc, Glucose; GalNAc, N-Acetylgalactosamine; GlcNAc, N-Acetylglucosamine; Fuc, Fucose. With kind permission from Springer Science + Business Media: Glycoconjugate journal, Norwalk virus-like particles bind specifically to A, H and difucosylated Lewis but not to B histo-blood group active glycosphingolipids, 26(9), 2009, 1172, Nilsson J, Rydell GE, Le Pendu J, Larson G, Figure 1 (the figure includes minor alterations).

However, as with most caliciviruses, understanding the interaction between RHDV and its host has been hampered by the lack of a suitable in vitro culture system. Consequently, studies on the pathogenesis of caliciviruses have relied on the ability of the capsid protein to self-assemble into virus-like particles (VLP) when expressed in insect cells. These particles have the advantage of being morphologically and antigenically indistinguishable from native virions, despite being devoid of viral RNA [59,101-105]. RHDV VLP strongly agglutinated human adult erythrocytes as the result of binding to glycolipid ligands present on the erythrocyte surfaces [72]. These ligands must develop with age since VLP did not agglutinate erythrocytes from human umbilical cords or foetuses presenting no agglutination [106]. Subsequent studies revealed that several caliciviruses use the carbohydrate moiety of host-cell histo-blood group antigens (HBGA) for attachment (e.g. ABH/O and Lewis antigens) initiating their replication cycle (Figure 2) [82,107-115]. Histo-blood group antigens are complex glycans either attached to proteins or lipids present on the surface of epithelial cells and erythrocytes, either as free oligosaccharides in biological fluids (milk, saliva, blood and intestinal contents). HBGA are formed by the sequential addition of monosaccharides to an oligosaccharide precursor chain attached to the cell glycans. This process, designated glycosylation, is catalysed by glycosyltransferase enzymes with specific substrate affinity and by a defined linkage [116]. Several genes encode the glycosyltransferases resulting in ABO, Lewis and secretor polymorphic phenotypes (Figure 3).

RHDV was shown to bind to the HBGA H type 2, A type 2 and B type 2 oligosaccharides [82,117]. These structures were shown to be present on the surface of the epithelial cells of the upper respiratory and digestive tracts that the virus first encounters when infecting the host and therefore where doors for virus entry are most likely located [16,82]. Synthesis of H type 2 requires the addition of a fucose residue, the minimal structural epitope [118], in α1,2 linkage to a precursor. This reaction is catalysed by an α1,2-fucosyltransferase which in rabbits is encoded by three functional genes, *Fut1, Fut2 *and *Sec1 *that have undergone multiple events of gene conversion during evolution [119]. Synthesis of the A and B type 2 antigens involves the addition of either a N-acetylgalactosamine or a galactose residue in α1,3 linkage to the H type 2 trisaccharide in a reaction catalysed by an α1,3-N-acetylgalactosaminyltransferase or an α1,3-galactosyltransferase (A or B transferases), respectively. In rabbits, the *ABO *blood group locus is largely unresolved, but preliminary data suggest that at least 6 *Abo *genes exist in the genome, arranged in tandem (K Nyström and J Le Pendu, personal communication).

Following the attachment of RHDV to the cell surface, internalisation, by an unknown mechanism, and desencapsidation occur, leading to the release of the viral genome into the cytoplasm. The virus life cycle then proceeds to the translation of the polyprotein precursor encoded by the ORF1 of the viral genome through interaction with the host cellular machinery. The gRNA and the sgRNA covalently-linked VPg uses the cellular translation machinery, positioning the ribosome at the initiation codon AUG without ribosome scanning and initiating translation [69,120]. Post-translational proteolytic processing by the viral gRNA encoded protease cleaves the polyprotein precursor into the mature non-structural proteins and, in RHDV, into the capsid protein VP60 [57]. The non-structural proteins, helicase and RdRp, then form a replication complex synthesising a complementary negative-sense RNA from the genomic RNA which is used as a template for the synthesis of gRNA and the sgRNA [reviewed in [121]]. The resulting RNA can either be de novo translated or packaged into viral particles that will be released from the infected cell. The mechanism used by RHDV for dissemination of the viral progeny is still unclear, but the ability of VP10 to induce apoptosis may suggest a role in programmed cell death in virion release and dissemination [70,95].

Translation of the RHDV ORF2 produces VP10 through a unique mechanism of reinitiation after termination of translation of the preceding major capsid protein [122]. This mechanism, although not fully understood, is dependent on the RNA sequence located upstream of the start/stop site, designated TURBS (termination upstream ribosomal binding site), but it is independent on the presence of the AUG initiation codon. Two critical motifs for VP10 expression have been identified within TURBS: motif 1, which is highly conserved among caliciviruses and shows complementarity with a short sequence in the 18S rRNA thus suggesting an interaction between the viral RNA and the ribosomal 18S rRNA, and motif 2, which is believed to be involved in the correct positioning of the ribosome at the translational start site [122,123].

## 6. Mechanisms of resistance to RHD

The lack of a cell culture system has been hampering the study of RHDV pathogenesis and, as a consequence, the mechanisms of resistance to the disease. The indirect strategies that have been employed for the study of the pathogenesis of RHDV allowed the identification of the HBGA H type 2 as an attachment factor for RHDV [82]. In humans, identification of alleles at the *ABO, FUT2 *and *FUT3 *loci that generate failure to express antigens recognised by different human *Norovirus *strains and that confer resistance to infection led to the search of such alleles in the α1,2-fucosyltransferase genes in adult rabbits [124]. This represented the first study on the genetic mechanisms underlying resistance to RHDV. A link between a rabbit allele at the α1,2-fucosyltransferase gene *Sec1*, that also intervenes in the H type 2 synthesis, and survival to a devastating RHDV outbreak was demonstrated [124]. This *Sec1 *allele encoded a weakly functional α1,2-fucosyltransferase, but was always found associated with *Fut2 *alleles coding for active enzymes that could compensate for the inability of Sec1 to synthesise H type 2. The authors hypothesised that this *Sec1 *allele was probably associated with a mutation located in the regulatory region of *Fut2 *which had compromised the Fut2 enzymes and, therefore, the synthesis of the virus' ligand. This result suggests that allelic variation in the α1,2-fucosyltransferase gene appears to have a significant role in resistance to RHDV. More recently, experimental challenge experiments indicated that at low virus titres, adult rabbits expressing low amounts of the HBGA ligands were less susceptible to the disease than animals expressing high amounts, although all animals were infected [117].

One striking characteristic of the pathogenesis is that of resistance of young rabbits less than 2 months of age to RHD [72]. Indeed, kittens less than 3 weeks old are fully resistant, but when infected at an age of 4 weeks or older the mortality rates increase to reach, at about 9 weeks old, the rates observed for adult individuals [10]. Thus, the mechanisms of resistance to RHDV have also been studied in light of the differences observed between adult and young rabbits. Interestingly, in young-resistant rabbits the attachment factor H type 2 has been shown to be weakly expressed on the epithelial cells of the upper respiratory and digestive tracts, where primary infection by the virus is believed to occur [82,106], which could explain their resistance to infection. Nevertheless, the reasons behind this differential expression have not yet been disclosed and the picture seems to be far more complicated. Indeed, and despite compelling evidence that supports a role of carbohydrates in facilitating infection by RHDV in epithelial cells of the upper respiratory and digestive tracts, other attachment factors or receptors must be playing a role at the epithelial level since low expression of the carbohydrate receptor at the doors of entry confers only partial protection against infection [117]. In addition, hepatocytes, the main cellular target for viral replication, have been shown not to express HBGA [82] and, in young rabbits, infection is accompanied by hepatic lesions due to virus replication as in adult individuals, although they tend to be more severe in 4 week old than in 2 week old rabbits [96,99,100,125,126]. This indicates the existence of at least additional hepatic cellular receptor(s) and that the genetic basis for the resistance mechanisms goes beyond the attachment of the virus to host cells through histo-blood group antigens. Immune response related-genes, either of the innate or the adaptive responses, are obvious candidate genes to be involved in the resistance mechanisms to RHDV and should deserve attention in future studies.

Differences in the innate immune response between RHDV-infected adult and young rabbits have also been observed [96,99,125-127]. Heterophils seem to be the predominant type of leukocyte in the liver inflammatory infiltrates in adult rabbits and are in close proximity with damaged hepatocytes probably being involved in the clearance of the dead cells. At variance, in young rabbits, this infiltrate is composed mostly by lymphocytes associated with undamaged and possibly antigen-presenting hepatocytes [127] and which are likely to mount a more effective and specific immune response than heterophils. In addition, in young rabbits only a small fraction of hepatocytes supports viral replication indicating that structural and functional changes have to occur in the liver to support RHDV replication [10,96,126].

Development of enzyme-linked immunosorbent assays (ELISA) for the diagnosis of RHD [29,35] allowed an early determination of the importance of humoral immunity in the course of the disease. Indeed, animals experiencing subacute forms of RHDV that survived infection and that were later resistant upon re-exposure to RHDV where shown to present high levels of seroconversion [35,72,128,129]. A correlation between cELISA titres and protection has been established which is important for determination of the applicability of vaccination and to assess the current status of the disease [54,128,130,131]. Recovering rabbits present IgM titres that quickly reach a maximum within 2 weeks and then sharply decrease. IgA titres are more prolonged in time, but they also face a decrease. In contrast, IgG slowly increase and are able to persist for months. With regards to IgA, this suggests a mucosal response [128]. Passive immunization (serotherapy) was also shown to be effective in stopping RHD in a rabbit farm hit by an outbreak [132], demonstrating the importance of humoral immunity in protection against RHD. In young rabbits, resistance has also been associated with the presence of maternal antibodies which are maintained during the period of life when they are considered RHD-resistant [128]. These are exclusively IgG acquired through the placenta in the last days of pregnancy and show a decline with age and body weight [19]. Additionally, if young rabbits are infected in their early life, they will become resistant when adult, suggesting that their immune system is capable of recognising the virus and producing an effective immune response that will confer long-term protection [133]. Therefore, humoral immunity clearly provides protection against RHDV when present [28,103].

## 7. Genetic diversity/RHDV evolution

### 7.1. Pathogenic RHDV

The origin and evolution of RHDV are not well understood. Although first reported in China in Angora rabbits imported from Germany, it was not clear if rabbits were already infected with RHDV when they arrived in China, since the disease might have been previously observed in Germany [73], or if they became infected later in China. The idea of RHDV being of Chinese origin has been challenged by several studies [50,134-136]. Indeed, these studies have shown that the pathogenic form of RHDV originated before 1984 [50,134,136] and that the Chinese strain isolated in 1984 had its origin in European isolates [135]. In light of these results, it seems that RHDV had its origins in Europe and that it had been circulating for some time, but that mortalities went unnoticed. Some hypotheses have been put forward regarding how RHDV originated. One of them, the transmission of the European brown hare syndrome virus to the European rabbit [137] has been discarded since EBHSV does not infect European rabbits. Other hypotheses propose that a virus from another species jumped to the rabbit where it became pathogenic [138], but the presently favoured hypothesis would be the change of a non-pathogenic virus closely related to RHDV and that rendered it pathogenic (see below).

Identifying novel features in the genome of RHDV might give some indications on the origin of the virus and its virulence. The first complete genome sequence of RHDV was obtained in 1991 by Meyers et al. [26]. The characterisation of the genetic diversity was initiated by sequencing and comparing partial sequences of a few European isolates [139-141]. The isolates were found to be highly similar and closely related.

Later, in an attempt to characterise the relation between EBHSV and RHDV, Wirblich et al. presented evidence that the N terminal portion of the capsid was highly conserved while the highest degree of variability was located in the C terminal half [39]. Indeed, while for the N-terminus homology between caliciviruses is ~80%, no strict conservation was observed for the C-terminus. In RHDV, this highly variable portion seems to correspond to the C and E domains as defined by Neill [47], where the majority of the differences between calicivirus isolates have been detected and where the main antigenic determinants have been found to be located [43,44,47,142-144]. These domains were predicted to be located at the capsid surface [46] and therefore more variable as a result of the strong selective pressure due to exposure to the host immune system.

**Figure 4:**
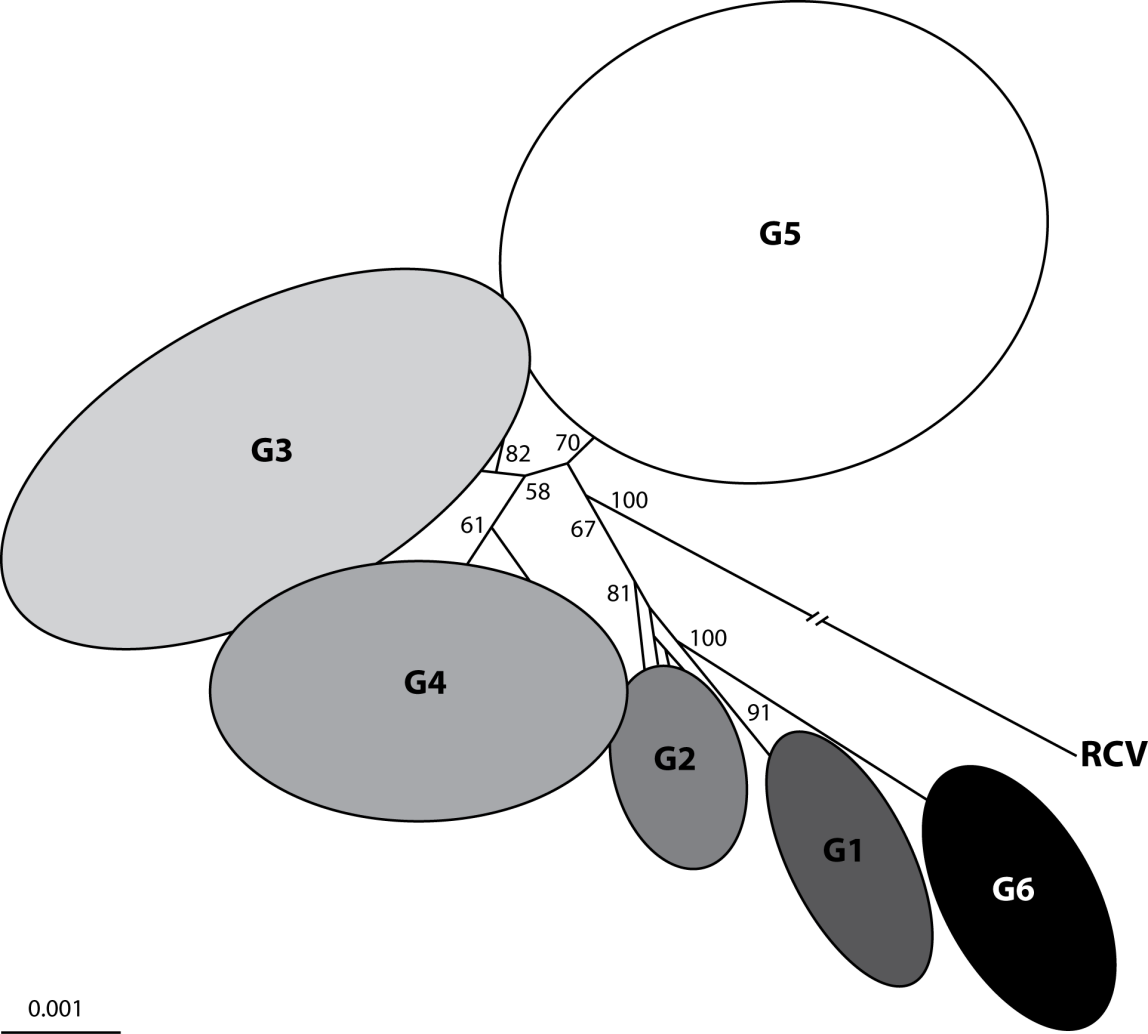
**Phylogenetic relationships between the RHDV genogroups G1-G6 and the Italian non-pathogenic strain RCV**. The tree was obtained using the neighbour-joining method and using nucleotide sequences from RHDV strains isolated worldwide. Bootstrap values greater than 50% are presented at the nodes. RCV was used as outgroup to root the tree. With kind permission from Springer Science + Business Media: Archives of Virology, Phylogenetic analysis of rabbit haemorrhagic disease virus in France between 1993 and 2000, and the characterisation of RHDV antigenic variants, 148(1), 2003, 72, Le Gall-Recule G, Zwingelstein F, Laurent S, de Boisseson C, Portejoie Y, Rasschaert D, Figure 2 (the figure includes minor alterations).

In 1997, the first phylogenetic analysis of RHDV isolates with different geographic locations and spanning the years from 1987 to 1995 was performed [137]. Inclusion of all the available information identified three major branches and supported the high degree of homology between samples, as previously reported, but also showed that RHDV strains clustered according to the year of isolation and not according to their geographic location. Le Gall et al. found the same pattern among French isolates, and further assigned the isolates into three chronologically established genogroups, G1, G2 and G3 [145]. Later, they observed that in France G1 and G2 had disappeared and three new genogroups had emerged: G4, having evolved from G3; G5, as a new independent group, and G6 (Figure 4) [14]. Interestingly, this genogroup G6 corresponded to the first antigenic variant of RHDV previously detected by Capucci et al. which they have designated as RHDVa [142]. This variant, although having the same level of pathogenicity as other RHDV isolates, presented a distinct antigenic profile and characteristic genetic differences [142]. Indeed, most of the amino acid variability found in RHDVa isolates was clustered in the 5' region of region E (spanning the amino acid positions 344-370), no reactivity was observed with the monoclonal antibody 1H8 that confers protection to experimentally-infected rabbits, but inoculation of vaccinated rabbits with RHDVa isolates caused no death [142,146]. RHDVa appears as a subtype of the RHDV wild-type (RHDVwt). These variants have been isolated in several countries and were detected as early as 1985 in China, where they might have emerged [13,136,146-150]. In some areas, these variants seem to be replacing the original strains [148].

In the Iberian Peninsula, only G1 strains have been found, even among contemporary strains [91,134]. This differs with the general pattern of RHDV evolution observed in other European countries, but, overall, G1 strains seem to evolve following a temporal rather than a geographic pattern as observed in the other genogroups [91]. This temporal structure is common in RNA viruses and likely results from the strong selection imposed by adaptive immune recognition by the host [151]. This was confirmed for RHDV, where a few positively selected codons were detected located in the major antigenic determinants of the capsid [49,50,134]. Interestingly, all the positively selected codons were associated with potential N-glycosylation sites. Glycosylation is known to play a role in the infectious process in other non-enveloped viruses such as rotavirus and Hepatitis E virus [152-155]. Positive selection recorded at N-glycosylation sites in the capsid protein of RHDV indicates that glycans might influence viral pathogenicity. This is further supported by the finding of non-pathogenic RHDV-like strains which do lack some of the positively selected N-glycosylation sites [49] although it is not known at present if the capsid protein from authentic virions is actually glycosylated.

Overall, with the exception of the RHDVa isolates, the evolution of RHDV is associated with a high degree of genetic homogeneity, with maximum nucleotide and amino acid differences of 10% and 6%, respectively [14,90,91,137,139-141,145,147,150,156-163], and mostly located in the regions C and E. These differences are much lower than those observed for other caliciviruses (e.g. for *Norovirus*, amino acid differences can reach a maximum of 61.4% while for *Sapovirus *they can reach 55%) [164,165]. This high homology may have resulted from the rapid spread of a new virus, expanding into a susceptible host population [139], but also from the fact that RHDV is a newly emerging pathogen whose evolution started recently, at variance from that of *Norovirus *and *Sapovirus*.

### 7.2. Non-pathogenic rabbit calicivirus

The emergence of RHDV from a pre-existing non-pathogenic rabbit calicivirus that has mutated and become pathogenic to rabbits has been hypothesised [136,139,160,166-168]. The detection of antibodies specific to RHDV in rabbit sera collected before the first RHDV outbreak, the identification of RHDV-seropositive rabbits where RHD was never recorded and the presence or persistence of viral RNA in populations where no overt signs of disease could be observed, provided compelling evidence for the pre-existence of a non-pathogenic RHVD-like virus in European rabbit populations [29,128,160,169-179]. This non-pathogenic virus would share antigenic properties with RHDV and circulated asymptomatically amongst rabbit populations before the first RHDV outbreak in China. Isolation of several non-pathogenic rabbit caliciviruses related to RHDV but with a tropism limited to the gut and no obvious pathogenicity further substantiated this hypothesis [160,166,168,176,180,181] and showed that the non-pathogenic strains are still circulating. Protection to RHD had been shown to be conferred by some of these non-pathogenic strains which might provide an explanation for the low level of RHD incidence in some regions of Australia and Britain [166,168,169,171,173,179,182-184]. More recently this hypothesis was confirmed experimentally when Strive et al. showed that the non-pathogenic Australian strain RCV-A1 is able to generate an antibody response that cross-reacts to RHDV and further protects animals from RHD, but not completely and not from infection [184]. Other strains, however, do not confer any kind of protection [181,185].

Understanding the evolutionary history and the origin of RHDV will benefit from further studies of the non-pathogenic strains. The isolation of non-pathogenic strains has so far already revealed striking differences between these strains and the pathogenic RHDV strains, with the most important being, beside pathogenicity, tissue tropism and capsid variability [160,166,168,176,180]. Interestingly, MRCV, a new variant of the non-pathogenic rabbit calicivirus (RCV)-like group that was recently characterised [180,186], displayed a pathogenicity of approximately 30% which is significant and in contrast with reports from other non-pathogenic strains. The viral RNA of this strain was detected in the liver rather than in the intestine which seems to indicate that the ability of a strain to cross the epithelia barrier and reach other organs such as the liver is an important feature for the emergence of the pathogenic forms and should be further explored. However, these results should be considered with caution since experimental reproduction of the disease failed, with only 2 out of the 14 inoculated rabbits presenting very mild symptoms.

Despite the accumulation of data, no mutations have yet been attributed that would explain the switch from a non-pathogenic to a pathogenic form. Recombination within the RHDV genome is not uncommon [187,188] and might have played a role in the origin of RHDV. Indeed, the recombination event reported by Forrester et al. [188] in the strain isolated in 1984 in China may suggest that recombination was a common mechanism at the time of emergence of the pathogenic forms. Nevertheless, not all pathogenic forms are direct descendants of this strain [135] and despite the relatively high frequency at which recombination occurs (4 out of 10), it does not seem to be widespread in all RHDV lineages [136]. This might be the result of an incomplete and non-systematic sampling or of the scarcity of complete genomic sequences.

Recently, novel phylogenetic analysis approaches were used to assess the emergence of RHDV [50,134,136]. Although the results could have brought an insight into the timing of the appearance of the non-pathogenic and the pathogenic forms of the virus, the studies led to incongruent results most likely as the result of the different capsid fragment lengths used in each study. This poses the question if the capsid is indeed the best gene for inferring RHDV history. While Kerr et al. could establish the Time to Most Recent Common Ancestor (TMRCA) between RHDV and the non-pathogenic forms of < 550 years and of < 150 years for the pathogenic forms [136], Kinnear and Linde set the existence of the ancestor of RHDV-RCV later in the 1930s and of the ancestor of the pathogenic RHDV strains between 1957-1976 [50]. Consistent with the findings of Kerr et al., Alda et al. set the TMRCA for all the RHDV to ~1884 [134]. Nevertheless, and considering these estimates, it is surprising that the disease had not been reported earlier than 1984. This might suggest an alternative scenario for the virus emergence where the virus would have come from another species through a species jump, that would have acted as a reservoir and was not affected by RHDV [136]. This scenario, however, implies the existence of such a reservoir host in which the virus was able to replicate. Viral RNA has been recently detected and isolated in micromammals living in sympatry with European rabbit populations that could represent the unidentified reservoir [94], but viral replication within these species could not be confirmed. Since these species might be important for virus transmission and spread and perhaps represent the "unknown" reservoir for when the virus seems to be inactive, i.e. between outbreaks, this hypothesis should be further explored.

## 8. Host-virus co-evolution

The virus-host dynamics result in a co-evolutionary process between the host resistance mechanisms and the virus escape mechanisms with attenuation of the virus and/or increase in resistance of the host. Therefore, the study of the host-virus co-evolutionary processes requires the analysis of each element of this dynamical pair simultaneously. Regarding the host, and in order to identify signatures of selection due to infectious agents, the natural history of this species should be considered. The fossil record suggests that the European rabbit originated in the Iberian Peninsula during the medium Pleistocene [189-192] and two morphologically differentiated subspecies have been distinguished: *O. cuniculus algirus *and *O. cuniculus cuniculus *[193]. *O. c. algirus *inhabits the southwestern Iberian Peninsula, while *O. c. cuniculus *is present in the northeastern Iberian Peninsula. These two subspecies diverged ~1.8 Mya [reviewed in [194]] and then, by a post-glaciation expansion from the southwestern refugium to North and from the Northeastern to South or West, a contact zone was established. While the natural populations of *O. c. algirus *remained confined to the southwest of the Peninsula, the natural populations of *O. c. cuniculus *later expanded its range north towards France, likely after the last glacial peak [195], where they still remain present. The expansion of these populations with successive bottleneck events caused a significantly lower genetic diversity of the wild French *O. c. cuniculus *populations compared to the Iberian populations [195-201]. The European rabbit gene pool has been manipulated by man through a recent single domestication event of French origin, and therefore, all domestic rabbits belong to the subspecies *O. c. cuniculus *[reviewed in [201,202]]. Today, by man-mediated dispersal, the subspecies *O. c. cuniculus *can be found in Europe, Australia, New Zealand, North and South America, and North Africa. The gene pool of the European rabbit populations worldwide have been shaped differently by these events and this might have also interfered with the resistance mechanisms. Therefore, when studying the co-evolution between the European rabbits and RHDV, these events should be taken into account.

The introduction of myxoma virus in Australia was soon followed by an increase in genetic resistance in rabbits and the appearance of less virulent strains [203]. Recent field studies conducted in Australia suggest that RHDV also has become less effective in keeping wild rabbit numbers low and that in some populations rabbit numbers are returning to the pre-RHDV levels [204]. Laboratory challenges have confirmed this scenario when inoculation of rabbits from different Australian populations with the original introduced strain (Czech strain V351) failed to induce mortality or induced mortality rates lower than that which was expected [205]. In addition, in an Australian population, the frequency of host resistant phenotypes, i.e., phenotypes that confer a weak binding of the virus to the host HBGA that facilitate infection and thus provide protection to the host, has significantly increased [117]. A similar co-adaptation process seems to be occurring both in New Zealand [92] and in rabbit populations from Europe [117]. Indeed, Nyström et al. found that in a French wild rabbit population recovering from a major RHDV outbreak the frequency of resistant phenotypes increased among the survivors [117]. As with the Australian population, the resistant phenotypes are associated with weak viral binding. This indicates that the virus has contributed to select resistant hosts in accordance to the binding specificities of the circulating RHDV strains and gives further support for a role of the HBGA in the virus epidemiology and suggests that the virus is shaping the hosts' HBGA diversity.

The virus also seems to be evolving to overcome the host resistance mechanisms since significant mortalities are still observed in the field, at least in Australia. Evidence for this comes from the fact that in comparative trials in rabbits known to be resistant to infection with Czech 351, modern field strains appeared more virulent than the original released strain, suggesting they had evolved to keep pace with changes in rabbit resistance [205]. In addition, HBGA specificities of the strains that evolved in France from 1988 to 2009 progressively shifted, allowing preferential recognition of subgroups of animals that express distinct HBGA motifs, which suggests an adaptation to the host genetic diversity [117].

## 9. Prevention, control and vaccination

In animals presenting subclinical or no clinical signs, passively acquired immunity has been shown to act successfully in emergency situations [132]. Indeed, this therapy, which is achieved by inoculation with a hyperimmune antiserum, confers short-term protection, preventing death. Nevertheless, passive immunization is ineffective on animals presenting clinical signs. Thus, as yet, no cure is available for RHDV-dying rabbits. Prevention and control of the disease through biosecurity and immunoprophylactic measures such as vaccination are, therefore, of utmost importance. Due to the lack of a cell culture system for efficient virus propagation, commercially available vaccines against RHDV are produced from tissue suspensions of experimentally infected rabbits, followed by chemical inactivation of the virus [132,177,206]. However, and to obviate the risks inherent to the manufacturing and use of this kind of vaccines (the use of infectious particles, the need for a safe disposal of contaminant residues, social concerns on animal welfare) the RHDV capsid protein has been tested in various studies as a subunit vaccine against RHD. Several heterologous expression systems or recombinant animal viruses have been developed to produce recombinant versions of the VP60 protein. The VP60 recombinant protein has been produced in *Escherichia coli *[140]; insect cultured cells [59,101-104]; yeast [207,208]; plants [209-213]; insect larvae [214] and recombinant animal-derived viruses [215-219]. Most of these systems were shown to be immunogenic and to confer protection against lethal doses of RHDV by eliciting a humoral response indicating that they are good substitutes for the tissue vaccines. Features such as low cost, high yields and ease of scaling up are amongst the most important factors for their commercial viability.

Although commercially available vaccines have proven effective in rabbitries, in wild rabbit populations vaccination campaigns are economically and logistically impracticable and their effects are considered insignificant [220]. Indeed, administration to wild rabbits implies capturing and handling of rabbits which by being a stress factor might increase the mortality rates [221]. In addition, this would need to be performed systematically since induced-immunity lasts no longer than 1 year [222] and efficacy has been shown to be dependent on several physiological parameters of the individuals [223]. Therefore, alternative approaches are being explored to overcome these limitations such as the development of vaccines with the capacity for horizontal transmission to ensure appropriate immunization of a relevant portion of the population [219], vaccines that may be administered by the oral or nasal routes [105,208,213,217,219,224,225] or the construction of bivalent vaccines [216,226]. Nevertheless, as yet, none of these vaccines has been registered or is commercially available.

Biosecurity measures for control and prevention of RHD, including surveillance, sanitation, disinfection and quarantine, are of high importance to limit propagation and to ensure prevention of the disease in particular in the rabbit industry. In countries where RHDV circulates in wild rabbits and where eradication is not achievable, these measures might prevent large-scale infection in rabbitries. Thus, a careful and correct management of the RHDV outbreaks is always dependent on the epidemiological situation of the regions where they occur. In addition, a continuous monitoring of the viral evolution in the field is fundamental for the quick detection of new genetic and antigenic variants which might be determinant for the application of the most appropriate measures.

## 10. Therapeutic applications of RHDV

More recently, RHDV VLP have been considered as a mean for cancer and pathogen immunotherapies [227-232]. The capsid protein of RHDV spontaneously assembles into VLP which are morphologically and antigenically indistinguishable from native virions, but devoid of the viral RNA [101]. By genetic engineering, RHDV VLP have been shown to efficiently incorporate antigens that might be presented to immune cells and to elicit an adequate cell-mediated and humoral immune response [228-232]. In addition, RHDV VLP have the advantage of being easily produced and at low cost, and, by deriving from a non-human virus, they are not susceptible to pre-existing neutralising antibodies [228], thus providing a reliable molecular tool for therapeutic applications.

RHDV has also been investigated for the study of virally-induced acute liver failure (ALF) in humans [233-239] as it fulfils several of the requirements to be a good animal model [233]. ALF is a condition characterised by severe liver injury, hepatic encephalopathy, coagulopathy and multiorgan failure, with viral infections (e.g. hepatitis A, B and E) and drug use (e.g. paracetamol overdose) amongst the commonest causes [240]. The hepatic lesions observed following infection by RHDV [99] resemble those caused by ALF in humans, but other physiologic, histological and biochemical alterations are also shared. Indeed, the hemodynamic changes, alterations in the intracranial pressure and histological alterations such as apoptosis observed in RHDV are common to ALF [74,233,241,242]. In addition, clinical symptoms such as prostration or convulsions observed on RHD are also observed in ALF [38]. RHDV has also been used for the study of therapeutic approaches for ALF [236,238,239].

## 11. Conclusions

Despite the lack of an appropriate cell culture system, some light has been shed on several aspects of RHDV and RHD. Nevertheless, the host-parasite interactions established between RHDV and the European rabbit are still unclear. Indeed, the role of some of the proteins encoded by RHDV is still unknown and the emergence of RHDV as a pathogenic form has not yet been resolved. In order to clarify these gaps, an effort should be made in obtaining full genomic sequences, including for non-pathogenic strains as these might contribute to understand the pathogenesis of RHDV. As for the host, and in particular as for key factors of susceptibility and resistance, the rabbit genome project should be considered and used for the study of candidate genes. By using temporal samples, i.e., samples of rabbits collected before and after RHDV outbreaks, one might determine those candidate genes. Further studies on immunity to RHDV and on the related non-pathogenic viruses are also warranted for a better understanding of the host-pathogen relationships. In addition, the study of the closely related EBHSV and its host, might contribute to the understanding of the interplay between lagoviruses and leporid species. The possibility that RHDV might be used as a model for the study of other calicivirus infections, in particular in view of its non-pathogenicity for humans, as well as for the development of anti-cancer and pathogen therapies transforms it into a valuable research molecular tool.

## 12. List of abbreviations

RHDV: rabbit haemorrhagic disease virus; RHD: rabbit haemorrhagic disease; EBHSV: European brown hare syndrome virus; EBHS: European brown hare syndrome; gRNA: genomic RNA; sgRNA: subgenomic RNA; VPg: virus genome-linked protein; ORF: open reading frame; RdRp: RNA-dependent RNA polymerase; DIC: disseminated intravascular coagulation; pi: post-infection; VLP: virus-like particles; HBGA: histo-blood group antigens; TURBS: termination upstream ribosomal binding site; RCV: rabbit calicivirus; TMRCA: Time to Most Recent Common Ancestor; ALF: acute liver failure.

## Competing interests

The authors declare that they have no competing interests.

## Authors' contributions

JA performed a study on the available literature on the subject, analysed the retrieved information and wrote the manuscript. WvdL, JLP and PJE revised the manuscript critically according to their own area of expertise. All authors read and approved the final manuscript.
